# Surrogate end points and their role in clinical trial

**DOI:** 10.4103/0253-7613.48880

**Published:** 2009-02

**Authors:** Jaykaran

**Affiliations:** Department of Pharmacology, Government Medical College, Majura Gate, Surat-395 001, India. Email:drjaykaran@yahoo.co.in

Sir,

I read the research article, Immunomodulatory effect of Tinospora cordiofolia extract in human immunodeficiciency virus positive patients' by Kalikar *et al*.[1] The authors have mentioned that 60% of the patients in the study group reported decrease in the symptoms, while 40% of the patients in the placebo group reported an increase in the symptoms. But there was no significant improvement in the CD4 count in the study group after six months.

This finding again raises the question of the validity of using surrogate end point in any study. Previous trials like CONCORDE also question the role of CD4 as a surrogate end point.[2] This is not the only example; there are other studies such as the use of ventricular premature beats to predict death from serious heart rhythm disturbance,[3] blood concentration of antibiotics to predict clinical cure of infection[4] etc., which reveal the problem associated with the use of surrogate markers. Surrogate end points are mainly used by pharmaceutical companies to reduce the sample size, duration and, therefore, cost of a clinical trial. Whenever surrogate markers are used in a trial, the following things should be assessed before drawing a conclusion about the results:[5]

1. The surrogate end point should be reliable, reproducible, clinically available, easily quantifiable, and affordable, and it should exhibit ‘dose response’ effects.
2. It should be a true predictor of disease. The relation between the surrogate end point and the disease should have biologically plausible explanation.
3. It should be sensitive; that is, positive result for the surrogate end point should pick up all or most patients at increased risk of adverse outcome.
4. It should be specific; that is, a ‘negative’ result should exclude all or most of those without increased risk of adverse outcome.
5. There should be precise cut off between normal and abnormal values.
6. It should have an acceptable positive predictive value; that is, ‘positive’ result should always or usually mean that the patient thus identified is at increased risk of adverse outcome.
7. It should have acceptable negative predictive value; that is, a ‘negative’ result should always or usually mean that the patient thus identified is not at increased risk of adverse outcome.
8. It should be amenable to quality control monitoring.
9. Changes in surrogate end point should rapidly and accurately reflect the response to treatment.
